# Association between maxillary sinus pathology and odontogenic lesions in patients evaluated by cone beam computed tomography. A systematic review and meta-analysis

**DOI:** 10.4317/medoral.23172

**Published:** 2019-12-24

**Authors:** Sonia Peñarrocha-Oltra, David Soto-Peñaloza, Leticia Bagán-Debón, José V. Bagán-Sebastián, David Peñarrocha-Oltra

**Affiliations:** 1 MD, Faculty of Medicine and Dentistry, University of Valencia, Spain; 2 DDS, MS, Master in Oral Surgery and Implant Dentistry, Faculty of Medicine and Dentistry, University of Valencia, Spain; 3 PhD, DDS. Associate Professor, Department of Stomatology, Faculty of Medicine and Dentistry, Valencia, Spain; 4 MD, DDS, PhD, FDSRCS. Professor of Oral Medicine, Faculty of Medicine and Dentistry, University of Valencia, Spain

## Abstract

**Background:**

A study is made of the association between maxillary sinus pathology and odontogenic lesions in patients evaluated with cone beam computed tomography.

**Material and Methods:**

A literature search was made in five databases and OpenGrey. Methodological assessment was carried out using the Newcastle-Ottawa tool for observational studies. The random-effects model was used for the meta-analysis.

**Results:**

Twenty-one studies were included in the qualitative review and 6 in the meta-analysis. Most presented moderate or low risk of bias. The periodontal disease showed to be associated with the thickening of the sinus membrane (TSM). Mucous retention cysts and opacities were reported in few studies. The presence of periapical lesions (PALs) was significantly associated to TSM (OR=2.43 (95%CI:1.71-3.46); I2=34.5%) and to odontogenic maxillary sinusitis (OMS) (OR=1.77 (95%CI: 1.20-2.61); I2=35.5%).

**Conclusions:**

The presence of PALs increases the probability of TSM and OMS up to 2.4-fold and 1.7-fold respectively. The risk differences suggests that about 58 and 37 of out every 100 maxillary sinuses having antral teeth with PALs are associated with an increased risk TSM and OMS respectively. The meta-evidence obtained in this study was of moderate certainty, and although the magnitude of the observed associations may vary, their direction in favor sinus disorders appearance, would not change as a result.

** Key words:**Sinus pathology, Odontogenic Sinusitis, Sinus membrane thickening, CBCT, Periapical lesions, Periodontal disease

## Introduction

Maxillary sinus pathology may be of rhinogenic, odontogenic, traumatic, allergic, neoplastic and bone-related origin (1). Alterations of the sinus mucosa secondary to dental disorders are a result of the close anatomical relationship between some teeth and the sinus floor (2).

In this regard, the upper molars and some premolars lie close to the floor of the maxillary sinus (3). Specifically, the closest lying tooth is the upper second molar, followed by the first molar (4). In addition, these teeth suffer a higher prevalence of periapical lesions (PALs) compared with other teeth, specifically on endodontic treated teeth (5), as well as greater susceptibility to periodontal disease due to furcation involvement (6).

Under normal conditions the abovementioned teeth are separated from the maxillary antrum by a dense cortical bone layer of variable thickness – though in some cases these structures are separated only by the mucoperiosteum (7). Such close proximity between the teeth and the maxillary sinus is associated to anatomical changes of the sinus membrane and to sinus radiologic opacities such as odontogenic maxillary sinusitis (OMS) and other disorders such as mucous retention cysts (MRCs) or retention cysts (RCs) (8). Thickening of the sinus membrane (TSM) is reportedly the most frequent alteration of the maxillary sinus, followed by MRCs and opacities (9).

Moreover, MRCs or RCs or antral pseudocysts (different pathological conditions but radiographically indistinguishable) presented a controversial etiology because they may or may not be associated with dental origin and periodontal infections (10).

Some authors consider the maxillary sinus to be normal in the absence of TSM, or when a uniform thickening of < 2 mm is observed (11). However, there is no agreement as to the threshold beyond which the thickness of the sinus membrane should be regarded as pathological.

Cone beam computed tomography (CBCT) has been recommended for preoperative evaluation of the available bone in the posterior maxilla and to assess the health or pathology of maxillary sinus in different dental medicine disciplines, and it provides three-dimensional images of maxillofacial structures, with negligible radiation doses compared to medical CT (12).

Although the causes underlying sinus diseases and their association to dental lesions remain subject to controversy, ear, nose and throat specialists consider that a dental origin should be considered in the presence of chronic sinusitis, though such explorations are rarely described in routine clinical practice (13).

In keeping with these observations, the primary aim of this systematic review was to evaluate the association between odontogenic lesions and the appearance of TSM and OMS in patients evaluated using cone beam computed tomography (CBCT). As secondary outcomes the periodontal disease status, the root proximity to maxillary sinus and the appearance of mucous retention cysts (MRCs) were considered in this regard.

## Material and Methods

- Study protocol

The present review was carried out following the Preferred Reporting Items for Systematic Reviews and Meta-Analyses (PRISMA) criteria (http://www.prisma-statement.org).

- Focused question

The review was made to answer the following focused question in Population, Exposure and Outcome (PEO) format (14): (P) Among dentulous or partially edentulous patients subjected to (E) CBCT evaluation, what relationship is there between odontogenic lesions and the appearance of (O) anatomical alterations of the sinus membrane, maxillary sinusitis and mucosal retention cysts?

Population features: The dentulous patients were considered as any patient that has teeth, and the partially edentulous patients as any patient that had loss at least one tooth in the posterior zone. Either dentulous or partial edentulous patients should present pathologies of odontogenic origin in the proximity of paranasal sinus cavities (e.g. periapical lesions of endodontic origin or apical periodontitis of endodontic origin, or unhealthy teeth, or periodontal disease, or tooth roots intruded or in tight relation with paranasal sinus).

Odontogenic lesions:

Periapical lesions of endodontic origin (PALs): Are those considered within the endo-periodontal lesions (EPL) terminology, according the recent world workshop of periodontal and peri-implant diseases and conditions on 2017 (15). The term endo-periodontal lesions describes a pathologic communication between the pulpal and periodontal tissues at a given tooth that may be triggered by a carious or traumatic lesion that affects the pulp and, secondarily, affects the periodontium, by periodontal destruction that secondarily affects the root canal; or by concomitant presence of both pathologies “true-combined” (15).

Periodontal disease: A patient is considered to have chronic periodontitis, if it is presenting a periodontal probing depth greater than 5 (PPD ≥ 5 mm ) and clinical attachment loss greater than 3 (CAL ≥ 3mm ) and angular bone loss ≥3 mm (16). The classification depends on additional measurements of the bleeding on probing values (BOP).

- Eligibility criteria

Inclusion criteria: Those randomized controlled trials, prospective or retrospective observational cohort studies, case-control studies, case series and cross-sectional studies that a priori assessed the TSM or OMS appearance in relation to an odontogenic origin in patients underwent CBCT imaging.

 Exclusion criteria: Systematic reviews, narrative reviews, nonclinical studies, *in vitro* studies, congress posters and abstracts, and case series involving fewer than 30 cases. Those studies failing to compile a priori information on TSM or OMS sinus pathologies were excluded. In the case of multiple publications based on the same patient sample, only the most recent data were considered.

- Electronic search 

Two reviewers (SPO and DSP) conducted an extensive but sensitive search of the main databases and grey literature in Medline via PubMed, EMBASE, the Cochrane Library, Web of Science (WOS), LILACS and OpenGrey (www.opengrey.eu). The search involved no language restrictions and extended up until September 2017. We used indexed terms, as well as free terms that were combined and adapted among the different databases. Lastly, the list of references of the included publications were evaluated in search of potential new articles (Table 1, Table 2). Discrepancies were resolved by discussion and consensus with a third consultant (LBD).

**Table 1:**
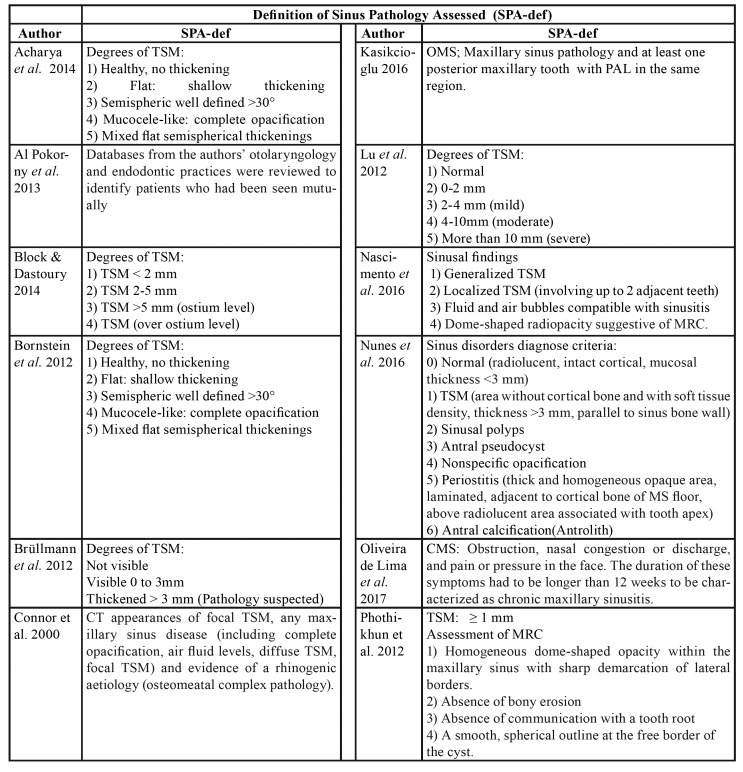
Diagnostic criteria to assess the sinus pathologies among included studies.

**Table 1 cont.**
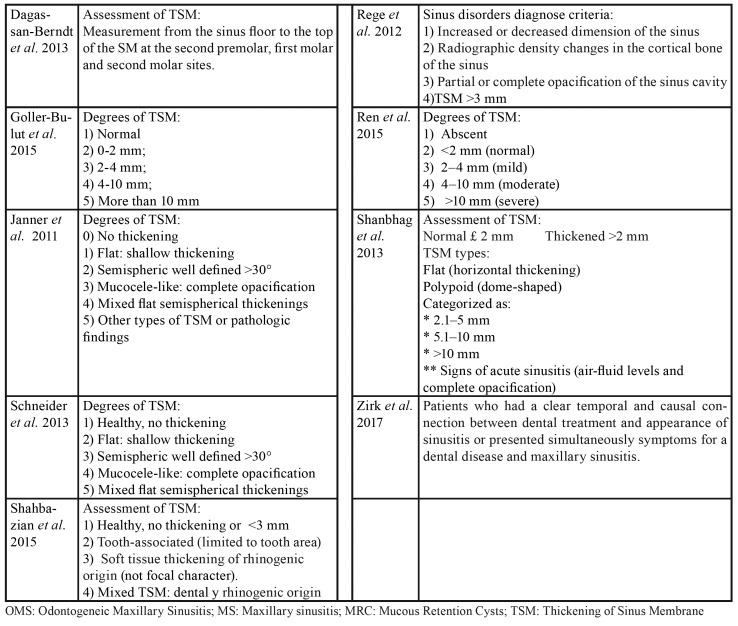
Diagnostic criteria to assess the sinus pathologies among included studies.

**Table 2:**
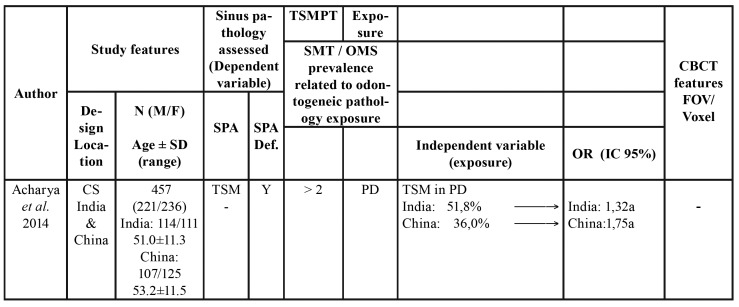
Characteristics of included studies.

**Table 2 cont.**
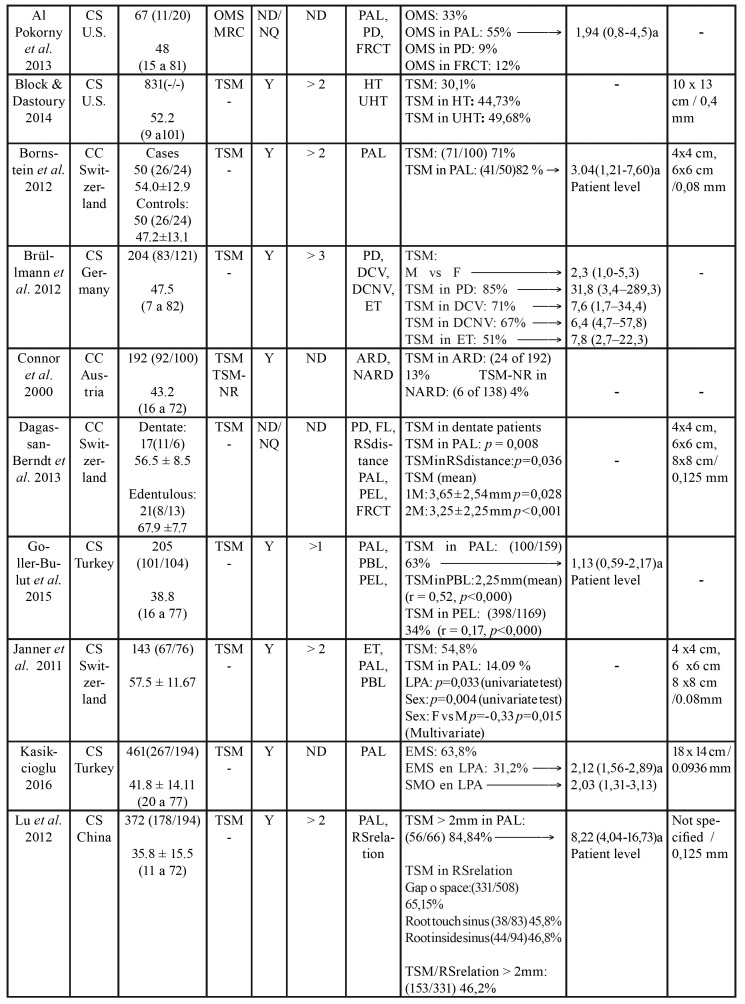
Characteristics of included studies.

**Table 2 cont.**
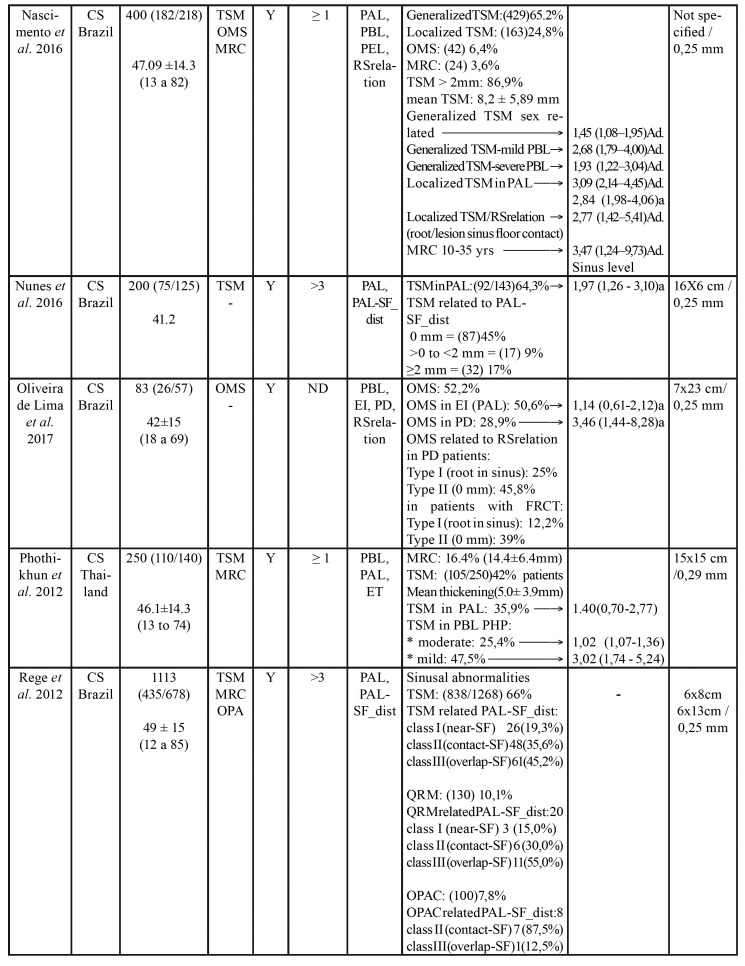
Characteristics of included studies.

**Table 2 cont.**
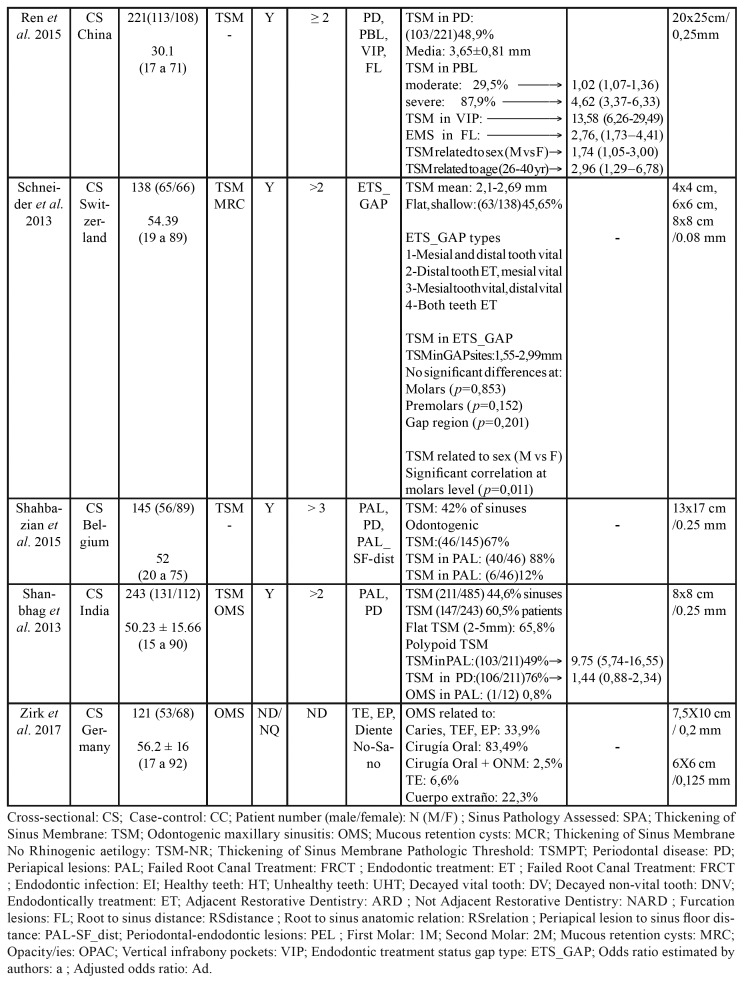
Characteristics of included studies.

- Study screening

After the elimination of duplicates, article selection by title and abstract was carried out independently by two reviewers (SPO and DSP). Full-text evaluation of the relevant articles was made applying the previously described inclusion and exclusion criteria. Interrater agreement was assessed by means of Cohen`s kappa coefficient (k). Discrepancies were resolved y discussion with an expert (DPO).

-Data extraction

Two reviewers (SPO and DSP) extracted a series of data to allow comparison and summarize the available evidence. The extraction process was performed in duplicate using an Excel® Table (Microsoft Office 2017, Redmond, WA, USA). The following data were extracted from the included studies: number of participants and gender, mean patient age or age range, sinus disease evaluated (dependent variable), definition of the threshold beyond which the thickness of the sinus membrane is regarded as pathological, odontogenic disease or condition related to the sinus alteration (independent variable), study objectives, material and methods (definition of sinus pathology), results, prevalence of TSM and OMS (%) in relation to the odontogenic disease or condition (independent variable), CBCT characteristics, and conclusions. Sinus pathologies :

TSM: It is considered as mucositis of the sinus membrane, normal sinus mucosa is not visualized on radiographs; however, when the mucosa becomes inflamed it may increase in thickness which may be seen radiographically. Thus TSM>2mm are considered as pathological sinus membrane inflammation (17).

OMS: Are those chronic rhinosinusitis of dental origin. Thickening around the entire wall of sinus mucosa and accumulation of secretions that accompany sinusitis reduce the air content of the sinus and cause it to become increasingly radiopaque (near or complete), mucosal thickening in just the base of the sinus may not represent sinusitis (10). The mucosa thickening is limited to the area of a tooth presenting one or more of the following conditions: caries, defective restoration, periapical lesion, periodontal disease or an extraction site (11).

MRCs: The term retention pseudocyst is used to describe several related conditions. The actual pathogenesis of these lesions is controversial; however, because their clinical and radiographic features are similar, no attempt is made here to distinguish them. One etiology suggests that blockage of the secretory ducts of seromucous glands in the sinus mucosa may result in a pathologic submucosal accumulation of secretions, resulting in swelling of the tissue. A second theory suggests that the serous nonsecretory retention cyst arises as a result of cystic degeneration within an inflamed, thickened sinus lining. Both types of lesions are called pseudocysts because they are not lined with epithelium (10). Retention pseudocysts usually appear as well defined, no corticated, smooth, dome-shaped radiopaque masses and no osseous border surrounds it.

- Evaluation of methodological quality (risk of bias)

The evaluation of methodological quality was carried out in duplicate and independently by two reviewers (SPO and DSP) using the Newcastle-Ottawa (NOS) tool for observational case-control studies (http://www.ohri.ca/programs/clinical_epidemiology/oxford.asp), which evaluates three aspects: “Selection”, “Comparability” and “Results”. The risk of bias was scored from 1-9 as follows: high (1-3), moderate (4-6) or low (7-9). Only the comparability dimension could obtain two points. An adaptation was used to assess the cross-sectional studies, affording two additional points to the definition of the disease. The discrepancies during this phase were resolved by consulting an expert (JVB). The kappa coefficient was used to assess concordance between reviewers, stratifying the level according to the Landis and Koch scale (18).

- Meta-analysis and certainty of meta-evidence

We calculated the odds ratios (ORs) for estimating associations between the prevalence of PALs and TSM and OMS. The data were obtained from the prevalence frequencies and percentages where possible. The global effect was quantified by means of a random effects meta-analysis. We estimated the corresponding Z-statistic, *p-value* and 95% confidence interval (95%CI). The estimations referred to OR (and log) were displayed by means of forest plots. Heterogeneity was assessed applying the Cochran Q test. The indicator I2 represents the degree of inconsistency of the results, with I2 values of 25%, 50% and 75% respectively indicating low, moderate and high heterogeneity. The precision of each study was evaluated based on Galbraith plots as an alternative to funnel plots, due to the limited number of studies available. If there is a study with outlier size effect introducing high heterogeneity, a sensitivity analysis is performed to test the robustness of estimation excluding the concerned study and repeating the analysis. The certainty of evidence is assessed trough the GRADE approach (as high, moderate, low or very low) by the integration of the risk of bias, inconsistency, indirectness, imprecision and other considerations through a summary of finding Tables (SoF), using the GRADEpro software (https://gdt.gradepro.org).

## Results

- Electronic search and study screening

**Figure 1:**
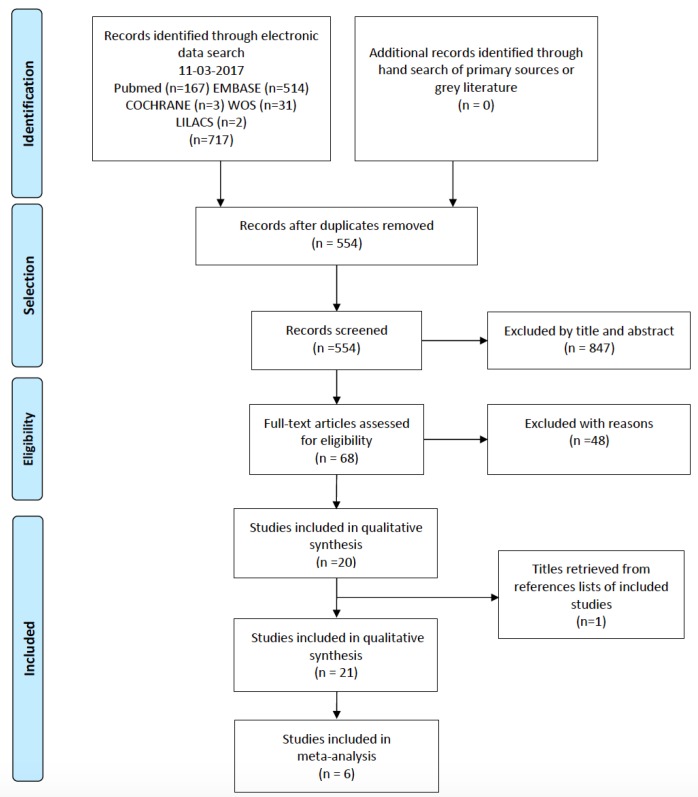
PRISMA flowchart of selection process.

The search of the main databases yielded 717 publications. After eliminating duplicates and evaluating titles and abstracts, a total of 67 studies underwent full-text evaluation, with the inclusion of 20 publications. One additional study was obtained by consulting the reference lists of the included articles. A total of 21 studies were therefore finally considered in the present systematic review. The PRISMA flow chart gives an overview of the article selection process (Fig. 1).

- Characteristics of the studies

The 21 selected articles comprised three case-control and 19 cross-sectional studies. Five were carried out in Brazil, four in Switzerland and the rest in other countries. Of the total studies, 16 evaluated TSM, two measured TSM and OMS (8,19), and four assessed only OMS (20-23). In addition to TSM or OMS, a number of studies evaluated other sinus disorders such as MRC (8,21,24,25) and opacities (9). Three studies offered no definition or quantification of sinus disease (4,21,23). Diagnostic criteria for sinus disorders reported among included studies are depicted in Table 1.

With regard to the threshold beyond which the thickness of the sinus membrane is regarded as pathological, three studies considered any thickening > 1 mm to be pathological (8,24,26), 8 studies established the threshold as > 2 mm (19,25,27-32), four as > 3 mm (9,33-35), and 7 studies offered no definition. In relation to the odontogenic disorders (independent variable) related to sinus disease (dependent variable), PALs were the most widely reported disorders (studied in 13 articles), followed by periodontal disease (described in 9 articles), endodontic treatment (described in 8 articles), and root proximity to the maxillary sinus and loss of periodontal bone (both reported in 5 studies). A total of 5984 patients were included in the present systematic review. A descriptive summary of the studies is provided in Table 2.

- Evaluation of methodological quality (risk of bias)

**Figure 2:**
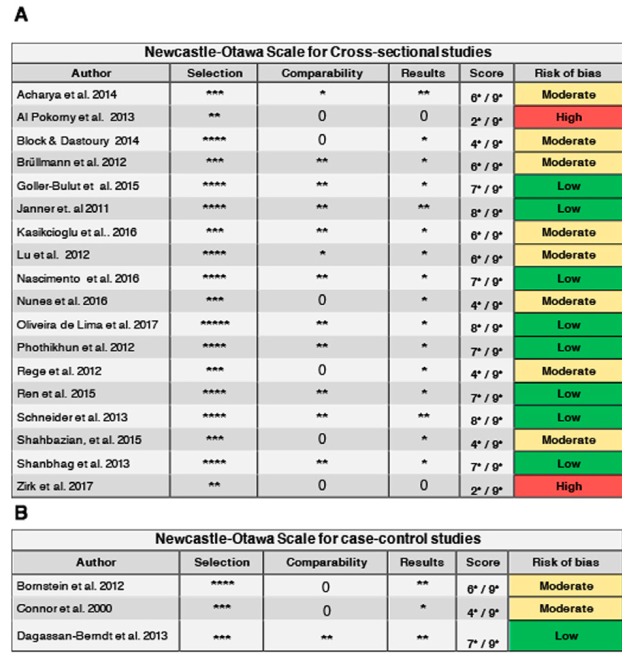
Summary of the risk of bias according study type. (A) Cross-sectional studies, (B) Case-control studies.

Inter-observer agreement during evaluation of the risk of bias was close to perfect according to the Landis and Koch scale (kappa k = 0.83). Moderate and low risks of bias were observed in the case-control studies, with scores of 4-7 out of the possible maximum of 9 (4,30,36). The least reported items were related to comparability, evaluated in a single study (4), and to the representativeness of the cases, due to demographic imbalances. Only one study failed to adequately report evaluator calibration during the tomographic evaluation process (36). Of the 19 cross-sectional studies, 7 showed moderate risk of bias, 9 low risk and three high risk. The score ranged from 3-8 (20,25,31) out of the possible maximum of 9. The least reported items were related to the selection of controls, implying the existence of selection bias in studies of this kind. All studies adequately defined sinus disease. The representativeness of the cases was inadequate in 8 studies. Regarding the comparability of the publications, 5 studies did not adjust the results to any relevant demographic or risk factor. The summary of risk of bias for either cross-sectional and case-control studies is depicted in Fig. 2.

- Qualitative synthesis

Prevalence of thickening of the sinus membrane and periapical lesions: Eleven studies evaluated PALs, and of these 7 identified an association between this variable and TSM (4,19,27,30,31,34,37). PALs grade was positively correlated to the prevalence and severity of TSM in posterior maxillary teeth, being more frequent in patients over 60 years of age in one study (27). Nascimento *et al*. (37) found the prevalence of localized TSM ≥ 1 mm to be 24%, versus 86.9% in the case of TSM > 2 mm. Likewise, TSM > 2 mm was associated to PALs, with ORs of 1.97 (34) and 9.75 (19), respectively – the latter being one of the estimates of greatest proportion in the available literature. Four studies reported no significant association, though they offered data on the prevalence of TSM (9,24,26,35).

Prevalence of odontogenic maxillary sinusitis and periapical lesions: Of the articles included in our review, 6 examined OMS in relation to dental disease (8,19-22,38), though only Kasikcioglu *et al*. found an association between maxillary sinusitis and PALs, with a significant OR of 2.03 (95%CI: 1.31-3.13). This relationship proved significant in relation to the posterior teeth, particularly the first and second molars (22). The rest of the articles that considered OMS offered no data regarding a possible association, though they did describe the prevalence of the disorder.

Thickening of the sinus membrane and periodontal lesions:

Of the 7 articles that examined the relationship between periodontal disease and TSM, five identified a positive association between them (19,24,26,28,32,33,37). The severity of periodontal disease as determined by moderate to severe periodontal bone loss was associated to TSM (24,28,32,37). One study observed a significant correlation between periodontal bone loss and a mean TSM of 2.25 mm (26). A single study, published by Dagassan-Berndt *et al*. (4), found no association between increased probing depth or the presence of furcal lesions and TSM. In two studies, the statistical significance of the association was lost on adjusting for variables such as patient gender and age (19), or in the multivariate analysis (31).

Root – maxillary sinus distance: The anatomical relationship between dental roots with odontogenic disease and the floor of the maxillary sinus was described in 6 of the included articles. Three studies reported a significant association between proximity of the diseased roots to the sinus and the prevalence of sinus disease (4,8,34). Oliveira de Lima *et al*. (20) found that the shorter the distance separating roots with endodontic infection from the maxillary sinus, the greater the risk of chronic maxillary sinusitis. In contrast, a 2.5-fold decrease in risk was observed as the mentioned distance increased (*p*<0.05). However, in two studies the spatial positioning of roots with periapical lesions was not seen to have an impact upon the prevalence of TSM (9,27).

Mucous retention cysts: Six studies reported the finding of MRCs in the tomography scans (9,21,24,25,37). Nascimento *et al*. calculated an OR of 3.47 for the presence of MRCs in the group of patients between 10 and 35 years of age versus those over 50 years of age. Rege *et al*. (9) found 10% of the patients with TSM to have MRCs, and of these, 26% were seen to be associated to teeth with PALs. Schneider *et al*. in turn observed MRCs in only 6 out of 49 maxillary sinuses (4.35%) (25). The remaining studies found no association between the presence of odontogenic disease and MRCs in the maxillary sinus.

- Meta-analysis

The quantitative synthesis was made by means of a random effects meta-analysis to assess the effect of PALs upon TSM, considering the number of maxillary sinuses as the analytical unit, with a total of 1505 sinuses. Furthermore, this odontogenic lesion was associated to the prevalence of OMS, analyzing a sample of 1190 sinuses. Information used for both meta-analyses subsets is provided in Table 3.

**Table 3:**
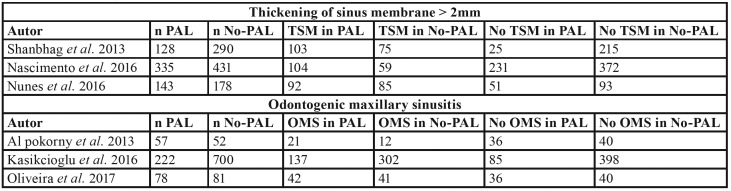
Data distribution employed for meta-analyses for the association between periapical lesion (PAL) presence and sinus pathologies (TSM>2mm and SMO).

**Figure 3:**
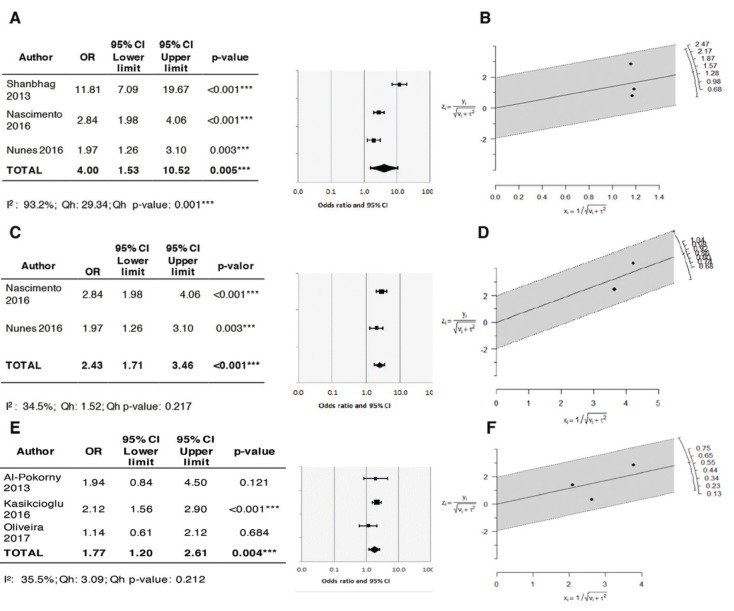
Forest plots and Galbraith´s plots to display heterogeneity for the association between PAL presence and the appearance of TSM > 2 mm and OMS. Global estimation PAL-TSM (A-B); Sensitivity analysis PAL-TSM (C-D); Global estimation PAL-OMS (E-F).

Association between PAL and TSM: All the studies included in the analysis reported a significant OR of over 1 for TSM > 2 mm, thus indicating that the presence of PALs increases the risk of TSM in comparison with the group without PALs (No-PAL). The study published by Shanbhag *et al*. (19) revealed a very strong correlation (OR=11.8), with introduction of great heterogeneity in the model. The estimated global effect in this meta-analysis yielded an OR of 4 (95%CI: 1.53-10.52) and I2=93.2%, with an interval excluding unity – thereby showing the association to be statistically significant (*p*=0.005) (Fig. 3). The Galbraith plots showed a study (19) that contribute to a great extent to the heterogeneity of the global estimate; this is situated more distant regarding the central axis compared the other two meta-analyzed studies (Fig. 3).

A sensitivity test was conducted for corroborating the consistency of the initial estimate, excluding Shanbhag *et al*. (19). Following the analysis, the global effect remained significant and less heterogeneity was observed, with an OR of 2.43 (95%CI: 1.71-2.46) (*p*<0.001) and I2=34.5% (Q=1.52; *p*=0.217). These results indicated that PALs could result in a 243% increase in the risk of TSM (Fig. 3). The Galbraith plots showed both studies to contribute similar heterogeneity to the global estimate (Fig. 3). No analysis of publication bias was made, since the number of studies entered in the meta-analysis was under 10 (39).

Association between PAL and OMS: The global effect estimated in this meta-analysis revealed a positive association between the presence of PALs and OMS, with an OR of 1.77 (95%CI: 1.20-2.61) and I2=35.5% (Q=3.09; *p*=0.212). The OR interval excluded unity – thereby showing the association to be statistically significant (*p*=0.004) (Fig. 3). The Galbraith plots show the distribution of the studies with respect to the central axis and the contribution to heterogeneity of the global effect (Fig. 3).

- Certainty of meta-evidence

The body of the meta-evidence is of moderate certainty for the outcomes assessed, the evidence was downgraded by 1 level due to the risk of confounding bias. Only data from sensitivity analysis was considered for TSM. The SoF Table, according to the GRADE approach is provided in Table 4.

**Table 4:**
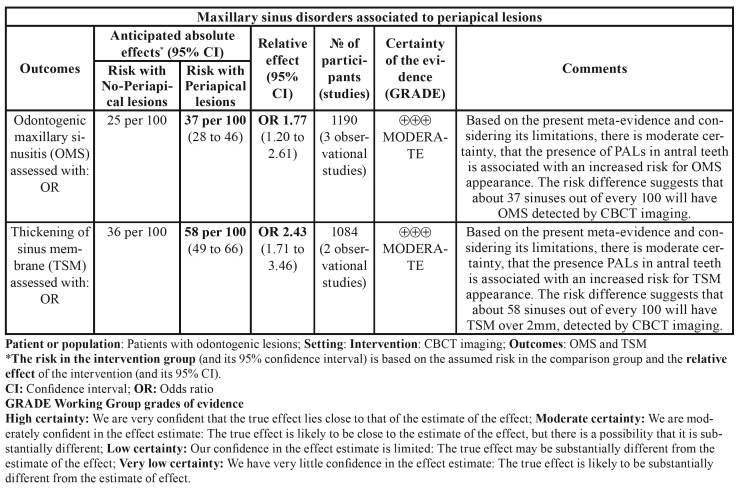
Summary of findings according to the GRADE approach.

## Discussion

The aim of this systematic review was to explore the possible association between pathology of the maxillary sinuses and odontogenic lesions in patients evaluated by CBCT.

Of the included publications, 16 evaluated TSM, two evaluated TSM and maxillary sinusitis, and four considered only maxillary sinusitis. Other sinus alterations such as MRCs (8,21,24,25) or opacities (9) were less frequently reported. Most of the studies described a positive association between the presence of periapical or periodontal lesions and alterations of the maxillary sinus (19,20,28,32,33). The prevalence of TSM in relation to PALs was variable, possibly because of the heterogeneity of the threshold defining pathological TSM (26,28,33) or the use of different tomographic resolutions (29,31).

Under normal conditions, the histologically measured thickness of the membrane ranged between 0.02-0.35 mm (38). However, when tomographic measurements were made, the mean thickness increased to 1.13 mm (39). This difference may be attribuTable to the imprecision of computed tomography in detecting measures < 0.5 mm or to contraction of the membrane as a result of fixation in formalin solution for histological study (40). Some authors define pathological membrane thickness as > 1 mm (8,24,26), while others establish the threshold from 2 or 3 mm (32,33,35).

Some contradictory results were observed with regard to the association between periodontal disease and sinus membrane thickness. In effect, a positive correlation was reported in 5 articles (8,26,28,32,33), while other studies found no significant association (4,19,31). One study initially identified a significant association, though statistical significance was lost on adjusting for factors such as patient gender and age. Nevertheless, the sign of the association did not change, and increased TSM continued to be observed in the presence of periodontal disease (19).

Other aspects addressed by the literature were closeness of the roots to the maxillary sinus (4,34) and mucosal retention cysts (8,9,21,24). Although some studies (9,27) reported no relationship between root-sinus distance and sinus disease, Oliveira de Lima *et al*. found the risk of OMS to decrease 2.5-fold as the tooth with endodontic infection was located further from the sinus (*p*<0.05) (20). On the other hand, Rege *et al*. reported a greater prevalence of MRCs in the presence of periapical lesions, with a prevalence of 10.1% (9). Similar data were reported by Bhattacharyya *et al*., with a prevalence of 12.4% (41). No cause-effect relationship has been demonstrated, however.

These associations can be explained in part by the fact that during extractions or in the presence of periodontal disease (e.g., periapical or endo-periodontal lesions, or loss of alveolar bone), teeth lying close to the maxillary sinus may damage the floor of the latter and even allow the spread of microorganisms of dental origin into the sinus (26).

Our meta-analysis revealed a significant association between PALs and TSM > 2 mm, on the basis of 1550 maxillary sinuses exposed to PALs (8,19,34). This association moreover remained significant and scantly heterogeneous after the sensitivity test, which confirmed the consistency of the estimation, with and OR of 2.43 (*p*<0.001), and I2=34.5% (Q=1.52; *p*=0.217).

On the other hand, of the articles considered in our review, 6 examined maxillary sinusitis in relation to dental disease (19-23,37) . It should be mentioned that the included studies did not confirm the diagnosis of maxillary sinusitis, since they only considered the radiological findings when the definition of maxillary sinusitis was fundamented on clinical and radiological criteria. Only the study of Oliveira de Lima *et al*. diagnosed sinusitis clinically, radiologically and by endoscopy performed by an ear, nose and throat specialist (20).

The meta-analytical estimate based on the CBCT study of 1190 maxillary sinuses exposed to PALs revealed a significant and positive correlation, in which the presence of such lesions was seen to imply a 1-7-fold greater risk of OMS than in the absence of sinus exposure to PALs. The evaluated studies had moderate methodological quality and did not show important demographic imbalances, with the exception of one publication that reported a 2:1 male-to-female proportion that may have led to underestimation of the association (20). Despite this, the analysis showed low heterogeneity, with accepTable confidence intervals.

Strengths and limitations:

The novelty of the present systematic review is that it conveys a broad perspective of an ancient topic, provides comprehensive summary of the different diagnostic criteria available for the evaluation sinus pathologies of odontogenic origin through CBCT, which could be described as the most understandable and complete summary that has ever been posted. The present study offers a first meta-analytical estimate referred to sinus disease, in particular the association between TSM and OMS, and the presence of PALs.

This acknowledged information was taken into consideration and integrated trough the GRADE approach to determine the certainty of meta-evidence in a transparent manner. The elaboration of this report summarize the best available literature, which does not mean is the less biased. Some limitations, such as the nature of the cross-sectional and case-control studies, with the presence of bias inherent to their retrospective design. Another relevant issue is confounding bias, since in the retrospective studies the relationship between prior exposure (disease or associated disorders) was not always adjusted to potential confounding factors that could have an impact upon the magnitude of the estimate.

Recommendations and generalizability:

It is strongly advisable to adopt data collection protocols allowing prospective evaluation of odontogenic sinus alterations, with a view to assessing their response to treatment, since retrospective studies are intrinsically unable to detect causal relationships. The results provided by this review are for utmost importance for clinicians of different medicine areas, in particular for those treating patients with persistent sinus pathology, and those facing regenerative procedures and implant therapy related to the posterior maxillary region. It is because was observed that postoperative sinusitis after sinus lift procedures is more frequent in patients with previous chronic sinusitis, and could be a significant cause of postoperative infection and implant loss (42). A foremost concern, since teeth undergone root canal treatment are more prone to be extracted than non-root filled teeth (43-45), and consequently possibly replaced with dental implants.

## Conclusions

Periapical lesions are associated to TSM and OMS, as evaluated by CBCT. The severity of periodontal lesions are associated to TSM. Other characteristics such as closeness of the roots to the floor of the maxillary sinus, or the presence of MRCs and opacities, are scantly reported and require further study. The presence of PALs is associated to an up to 2.4-fold greater risk of TSM compared with sinuses not exposed to PALs and could result in a 243% increase in the risk of TSM. There is a positive correlation between PALs and OMS, with a 1.7-fold greater risk of suffering sinusitis in the presence of PALs than in their absence. The risk differences suggest that about 58 and 37 of out every 100 maxillary sinuses having antral teeth with PALs are associated with an increased risk TSM and OMS respectively. Based on the appraised meta-evidence and considering its limitations, there is moderate certainty, that the presence of PALs in antral teeth are associated with an increased risk for TSM and OMS appearance as evaluated by CBCT, and although the magnitude of the observed associations (quantitative interaction) may vary, their direction in favor sinus disorders appearance, would not change as a result.
